# Short-term cell death in tissues of *Pulsatilla vernalis* seeds from natural and ex situ conserved populations

**DOI:** 10.1038/s41598-021-95668-2

**Published:** 2021-08-19

**Authors:** Katarzyna M. Zielińska, Andrzej Kaźmierczak, Ewa Michalska

**Affiliations:** 1 Department of Geobotany and Plant Ecology, Faculty of Biology and Environmental Protection, University of Lodz, Banacha Str. 12/16, 90-237, Lodz, Poland; 2 Department of Cytophysiology, Faculty of Biology and Environmental Protection, University of Lodz, Banacha Str. 12/16, 90-237, Lodz, Poland

**Keywords:** Plant ecology, Physiology, Plant sciences, Conservation biology

## Abstract

*Pulsatilla vernalis* is a IUCN listed species that occurs in mountain and lowland habitats. The seeds collected from different populations are remarkably diverse in their viability depending on locality or year of collection. We aim to analyse seed viability, among others, by investigation of the percentage of alive, dying, and dead cells in embryos and endosperm when comparing the seeds from a wild lowland population and ex situ cultivation of plants of lowland and Alpine origin. The cell death was detected by staining with two fluorescence probes, one penetrating only the changed nuclear membranes, the other penetrating also the unchanged cells. 54.5% of Alpine origin seeds were presumably capable of germination if they were sown after collection, however, four months later only 36.4% had healthy embryos. In the case of lowland wild plants it was 31.8% and 18.2%, and from ex situ, 27.3% and 13.6%, respectively. 27.3% of Alpine origin seeds had embryo in torpedo stage (9.1% in the case of lowland seeds). Mean weight of the former was 2.9 mg (2.0 mg in lowland ones). Our results confirm the significance of seed origin and seed weight on viability, and that *Pulsatilla* seeds have a short ‘germination time window’.

## Introduction

**Figure 1:**
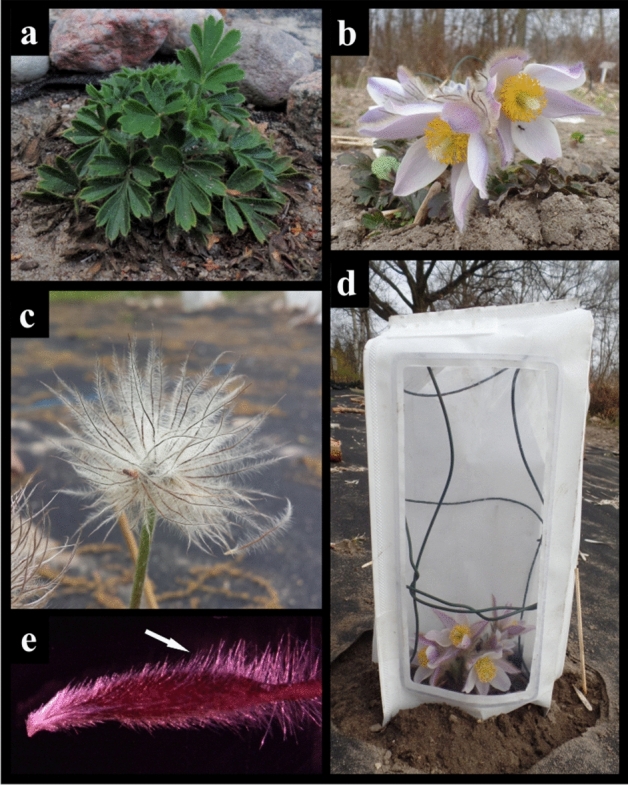
Specimens of *Pulsatilla vernalis* (L.) Mill. growing in ex situ cultivation in the Botanical Garden in Lodz: (**a**) a specimen with summer leaves (25.07.2019), (**b**) a flowering specimen (25.03.2019), (**c**) collective fruit (13.05.2019), (**d**) a specimen covered with the bag from Duraweld material (25.03.2019), (**e**) seed coat with characteristic trichomes.

*Pulsatilla vernalis* (L.) Mill. (spring pasque-flower), also known as *Anemone vernalis* L. (Fig. 1), is a species strongly threatened with extinction in European Lowlands^1–3^. At the turn of the nineteenth and twentieth centuries it was found in scattered but numerous lowland localities^3^, these localities have been disappearing through the twentieth century up till now, and the pasque-flower has been entered into the IUCN Red List^3–5^. The lowland localities are considered a relic of the past climatic oscilations^3,6,7^. The species originated from the Alpine chain and migrated down to lowlands in cold climate, thus *P. vernalis* is one of the key species for the analysis of plant post-glacial migration routes^6^, and adaptations that allow species to survive in the face of climate change.

The issue of saving lowland populations of *Pulsatilla vernalis* by their reinforcement, which encompass germination of seeds collected from natural populations and planting of the obtained seedlings, was taken up by Betz and his research team^8^ in Germany and by Nawrocka-Grześkowiak and Frydel^9^ in Poland. Our preliminary studies involving germination of seeds from different lowland populations demonstrated great variability of their viability depending on the year and locality of collection (Appendix 1).

Since the quality of the seeds may depend on the conditions in which the mother plant grew^10,11^, it could be expected that those obtained from ex situ cultivation, where plants do not have to compete for resources and are protected against unfavorable conditions (e.g. they are watered when rainfall is too low), should have high viability^12^. In this research, the condition of seeds collected from ex situ plantation and from natural site was analyzed using methods allowing to assess the viability of nuclei of embryo and endosperm cells. The method is based on the fact that membrane permeability increases^13,14^ in dying cells. With the use of two fluorescence probes, one penetrating the nuclear membrane only when it is changed (ethidium bromide) the other penetrating also the unchanged cells (acridine orange), the degree of membrane permeability or its damage can be detected^15^. The recorded percentage of alive, dying, and dead cells allows determination of the condition of embryo and endosperm, and, hence, if the seeds were potentially germinative.

In *Pulsatilla vernalis*, morphological dormancy occurs (MD)^16^, i.e. during dispersion seed embryos are differentiated into cotyledons and hypocotyl-radicle but underdeveloped in terms of size^17,18^. These embryos need time to grow before germination, and while the embryo increase in size, endosperm is gradually obliterated. It could be presumed that if the embryo cells are alive and the endosperm shows signs of dying, it means that the mentioned processes have started. However, the presence of dead cells should not be interpreted in such a way. In most cases, the final stage of cell death is related to a gradual nucleus degradation^19^. In our study, dead cells often cannot be identified, because the method used is based on the nuclei staining. Such a phenomenon was also observed during cell death-dependent aerenchyma formation when nuclei are degraded before the cell wall degradation^14^. Consequently, the lack of dead cells proves that only endogenous parameters are responsible for the physiological state of the seed.

**Figure 2:**
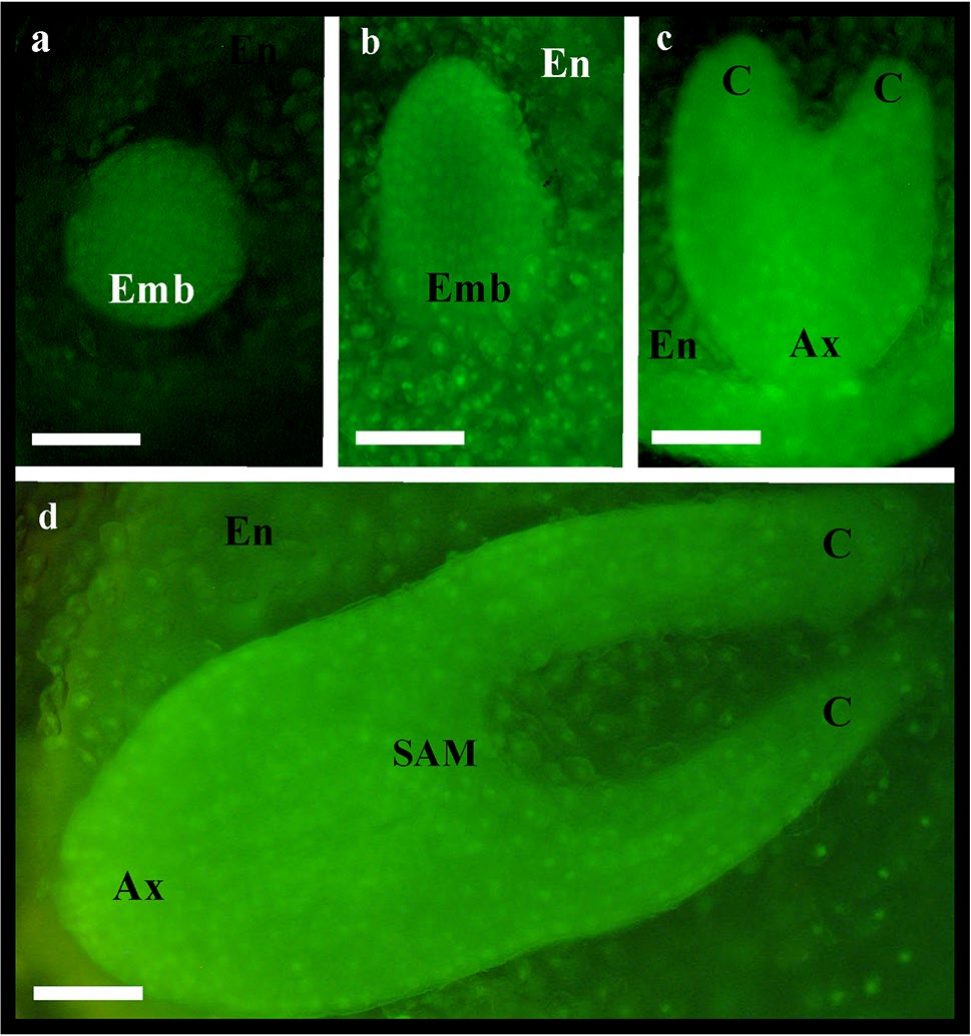
Globular (**a**), globular-heart transition (**b**), heart (**c**) and torpedo (**d**) of embryo developmental stages of *Pulsatilla vernalis* seeds noted under the fluorescence microscopy after staining with ethidium bromide and orange acridine. Scale bars = 100 µm. Ax, axis; C, cotyledon; Emb, embryo; En, endosperm; SAM, shoot apical meristem.

It seems that MD-seeds do not show crucial physiological sensitivity to environmental conditions, however, these conditions can influence the speed of embryo development and seed coat permeability^20^. The seeds of *P. vernalis* germinate in the same year, sprouting in the spring of the following year is very rare^21,22^. This means that the proper rate of embryo development is crucial, and this can be influenced by environmental conditions. During the seven years of research on Polish lowland population near Rogowiec, emergence of numerous seedlings was noted only once and it was related to the weather conditions^22^. It seems that successful reproduction strictly depends on favorable conditions occurring just in the right time. There is a small ‘time window’ for the seed to develop before it degenerates. It can be assumed that proper embryo development (cotyledons and hypocotyl-radicle occurrence i.e. torpedo stage) in the dispersed seeds is crucial for their successful germination. Thus, the comparison of the seeds from ex situ cultivation and from natural sites in regard to the degree of embryo development was a very important part of the work (Fig. 2).

It should be emphasized that the low percentage of germinating seeds from lowland localities (Appendix 1) can be the effect of loss of genetic diversity in the relatively small, declining populations. In the case of rare species, the size of population is frequently linked with reproductive fitness^23–26^. The same was found in the case of *Pulsatilla vulgaris* Mill. in Central Germany: mean seed set, seed number, and mean seed mass per population were attributed to differences in population size^27^. The analysis of genetic drift/inbreeding effects on the seed quality is beyond the scope of this study, however, it can shed light on the issue thanks to the comparison of seeds of different origin. We compared seeds from plants growing in the same conditions (ex situ): ones of Polish origin (where we deal with lowland small populations) to the ones of Alpine origin (where the pasque-flower is in its core range).

In *Pulsatilla*, the procedure of seed collection for ex situ germination requires quick assessment of their quality. It is widely recognized that there is a likely correlation between seed weight and its morphometric features, and the germination percentage and seedlings quality^28^. Thus, the analysis of the relationship between seed viability and their weight and length was important for the the results of the paper.

This article aimed at explaining the very diverse germination success of pasque-flower seeds of different origin. We analyzed three samples of seeds: (i) those collected from the natural lowland site, (ii) those from specimens growing in ex situ culture that were obtained from the seeds from the mentioned lowland site – Polish origin, and (iii) from specimens in ex situ culture obtained from the seeds of Alpine origin. We compared these seeds in respect of: (1) the percentage of alive, dying ones in the first or in the second stage of the process, as well as of the dead cells in embryo and endosperm; (2) embryo development stages; (3) the chosen biometrical features such as weight and length.

## Results

**Figure 3:**
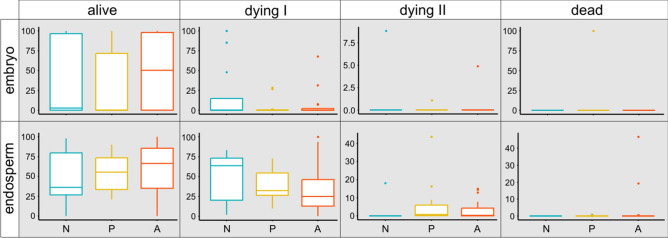
The share of alive, dying (in first and second stage) and dead cells noted in endosperm and embryo of *Pulsatilla vernalis* seeds, which were not undeveloped: 12 seeds of natural origin (N), 15 seeds from ex situ plantation of lowland Polish origin (P) and 22 of Alpine origin (A); boxplots with median, interquartile (Q1-Q3) range, min–max and outliers were made for the values of percentage share of alive, dying and dead cells in the seed preparation.

**Figure 4:**
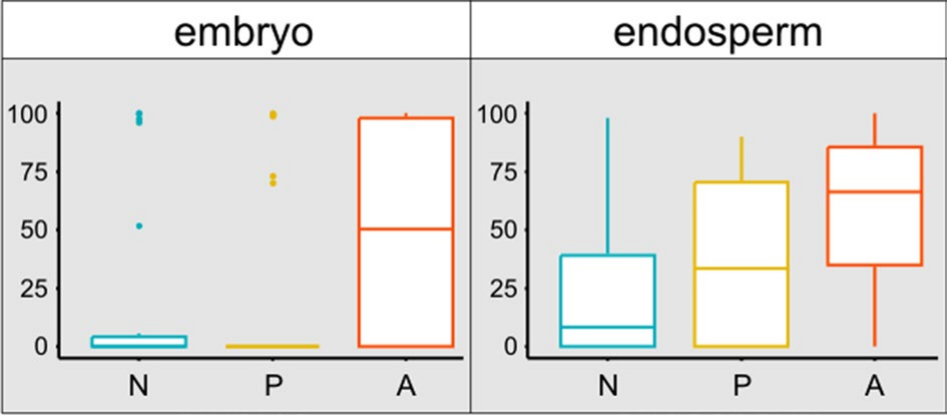
The share of alive cells noted in endosperm and embryo of *Pulsatilla vernalis* seeds counted on the samples of 22 seeds collected from natural site (N), and from ex situ plantation of Polish (P) and Alpine (A) origin; boxplots with median, interquartile (Q1-Q3) range, min–max and outliers were made for the values of percentage share of alive cells in the seed preparation.

Three groups of seeds, each containing 22 ones: that of natural origin (N), and from plants in ex situ plantation of Polish (P) and Alpine (A) origin, significantly differed in terms of their viability. A large number of totally undeveloped seeds is noteworthy. As many as 10 N seeds and 7 P seeds were empty (they had only seed coats), only in the case of A the whole set of 22 seeds was taken for laboratory tests. The latter ones also have the superior viability reflected by the percentage of alive cells in embryo and endosperm structures (Fig. 3). Importantly higher quality of the seeds of Alpine origin is even more evident in the statistics for equal samples of 22 ones (empty seeds were counted as the ones with the number of cells equal to zero) (Fig. 4). The data concerning the counting of alive, dying and dead cells, and standard errors are attached in Appendix 2.

According to the presented statistics, the number of alive cells is much bigger than of dying and dead ones, however, it must be stressed that it does not mean that there are many healthy seeds which are capable of germination (Table 1). We found four healthy seeds, with alive embryo and endosperm, which most likely would be able to survive winter and germinate in the spring of the following year. In that group, the seeds with embryos at the torpedo stage, which means that during dispersion they were fully developed and prepared for dormancy, would have the highest chance for germination. Only two such seeds (of Alpine origin) were found (Table 1, deep dormancy category).

**Table 1:**
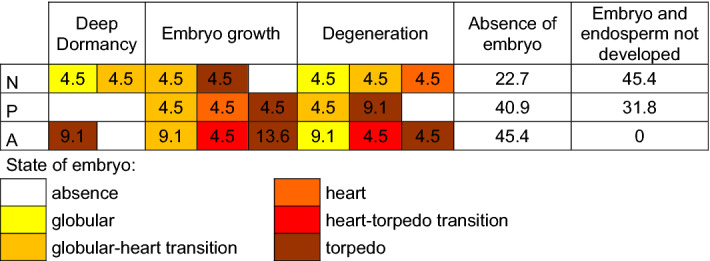
The state of seeds of natural origin (N), from ex situ plantation of plants of Poland (P) and of Alpine origin (A); the numbers represent percentages of seeds with embryos at the particular stage; alive seeds at ‘deep dormancy’ stage mean the embryo and endosperm with > 95% of alive cells; ‘embryo growth’ means the seeds with alive embryos and partly dying endosperm; seeds during ‘degeneration’ means those with more than 5% of dying embryo cells.

The seeds with healthy embryos and partly dying endosperms may be those whose deep dormancy was broken or those that were damaged by adverse environmental conditions. Since all the seeds before the analysis were stored together at room temperature, substantial differences in their state are most probably produced by internal causes. This was confirmed also by our preliminary studies which showed no damage of the seed coats in the stored seeds (using Evans blue staining method, Appendix 1) and the fact that completely dead cells were observed very rarely. We found 11 seeds with healthy embryos and partly dying endosperm, five with embryos at torpedo stage (Table 1, ‘embryo growth category’).

A big number of the analyzed seeds was clearly degenerated, we found 10 with a significant number of dying cells in embryos and a varied number of such cells in endosperm. The stage of embryo development varied from globular to torpedo (Table 1, ‘degeneration category’). In many cases, the embryo or the whole inner structure of a seed was absent, such was the case with 68% of N,73% of P and 45% of A seeds (Table 1). The data concerning embryo development stage of particular seeds are attached in Appendix 2.

**Figure 5:**
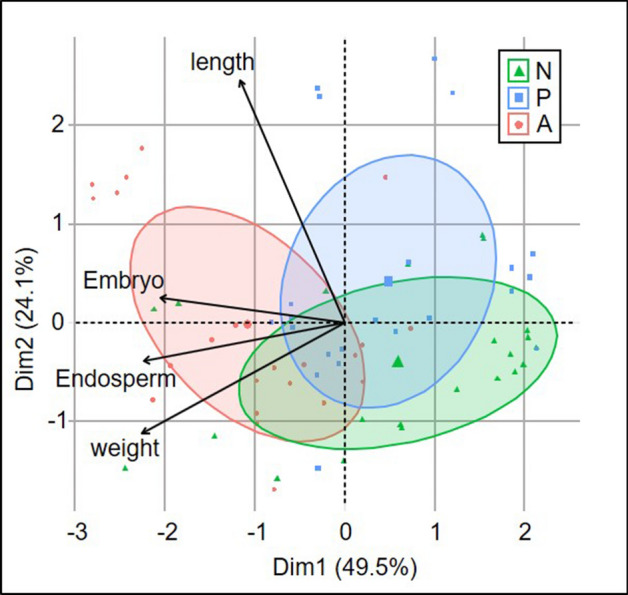
Principal component analysis (PCA) for the data concerning the share of alive cells in embryo and endosperm, and the biometric features of seeds, the three samples of 22 seeds collected from natural site (N), and from ex situ plantation of Polish (P) and Alpine (A) origin are marked.

PCA analysis confirmed that the seeds of Alpine origin differed significantly from those from Poland (Fig. 5). Comparing Polish seeds from the natural site and ex situ cultivation, we found that the former were more differentiated in terms of the percentage of alive cells and weight. It should be noted that the vector showing the share of viable cells in embryo is quite close to the vector of viable cells in endosperm, they are also correlated with the weight of seeds (Fig. 5). The data concerning weight and length of seeds are attached in Appendix 2.

According to the model based on multiple stepwise regression, the percentage of alive cells in embryo importantly grows with the seed weight, to a lesser extent with length and the number of dying endosperm cells at the second stage of that process (Table 2). The same analysis of the data from 49 seeds that were taken to laboratory analysis (crossection did not show that the seed is undeveloped) gave almost the same results, however, weight was important with *p* = 0.007, length with *p* = 0.02, percentage of dying endosperm cells with *p* = 0.009, and the quality of model was much lower (*p* = 0.005).

**Table 2:** Variables significantly dependent on the share of alive cells in embryo according to the model obtained by multiple stepwise linear regression on the basis of 66 data set, residual standart error: 36.04 on 62 degrees of freedom, F-statistic: 10.92 on 3 and 62 DF, *p* value: 7.466e-06, significance codes: ‘***’ 0.001 ‘**’ 0.01 ‘*’ 0.05.

Step regression procedure		Df	Sum of Sq	RSS	AIC
Endosperm alive cells		1	20.2	78,021	478.96
+ Endosperm dead		1	731.8	78,733	479.55
+ Endosperm dying I		1	1295.3	79,296	480.03
+ <None>				78,001	480.94
+ Length		1	6022.9	84,024	483.85
+ Weigth		1	12,494.1	90,495	488.74
+ Endosperm dying II		1	14,957.4	92,958	490.52
Endosperm dead		1	775.7	78,797	477.61
+ Endosperm dying I		1	1957.7	79,979	478.59
+ <None>				78,021	478.96
+ Length		1	6590.1	84,611	482.31
+ Endosperm dying II		1	15,130	93,151	488.65
+ Weigth		1	25,201.7	103,223	495.43
Endosperm dying I		1	1721.9	80,519	477.03
+ <None>				78,797	477.61
+ Length		1	6614.8	85,412	480.93
+ Endosperm dying II		1	14,358.9	93,156	486.66
+ Weigth		1	24,709.3	103,506	493.61
<None>				80,519	477.03
+ Length		1	6850.8	87,369	480.42
+ Endosperm dying II		1	12,766.3	93,285	484.75
+ Weigth		1	23,954.4	104,473	492.22

Summary		Estimate	Std. error	t value	Pr( >|t|)
(Intercept)		− 55.9841	16.9731	− 3.298	0.00161**
+ Weigth		24.8324	5.782	4.295	6.27E−05***
+ Length		5.7219	2.4913	2.297	0.02502*
+ Endosperm dying II		2.0766	0.6623	3.135	0.00262**
Residuals:	Min	1Q	Median	3Q	Max
	− 79.788	− 25.58	− 1.563	19.24	71.527

The dependence of the type of embryo was checked with the same method and the results were also similar: weight, length and dying endosperm cells (at the second stage) were positively linked with the degree of embryo development (Table 3). The analysis of the data concerning 49 seeds gave similar results – the same as in the previous case. Weight was important with *p* = 0.009, length with *p* = 0.08, percentage of dying endosperm cells with *p* = 0.002, and the quality of model was lower (*p* = 0.005).

**Table 3:** Variables significantly dependent on the stage of embryo development according to the model obtained by multiple stepwise linear regression on the basis of 66 data set, residual standard error: 1.303 on 62 degrees of freedom, F-statistic: 11.44 on 3 and 62 DF, *p* value: 4.557e-06, significance codes: ‘***’ 0.001 ‘**’ 0.01 ‘*’ 0.05 ‘**.**’ 0.1.

Step regression procedure:		Df	Sum of Sq	RSS	AIC
Endosperm alive cells		1	0.0384	100.47	39.731
+ Endosperm dying I		1	1.3517	101.78	40.588
+ Endosperm dead		1	2.969	103.4	41.629
+ <None>				100.43	41.706
+ Length		1	4.617	105.05	42.673
+ Weigth		1	15.5149	115.94	49.187
+ Endosperm dying II		1	28.5646	128.99	56.227
Endosperm dying I		1	2.128	102.59	39.115
+ <None>				100.47	39.731
+ Endosperm dead		1	3.106	103.57	39.741
+ Length		1	5.133	105.6	41.02
+ Endosperm dying II		1	28.894	129.36	54.414
+ Weigth		1	31.696	132.16	55.829
Endosperm dead		1	2.6086	105.2	38.772
+ <None>		1	5.366	102.59	39.115
+ Length		1	26.7657	107.96	40.479
+ Endosperm dying II		1	30.9391	129.36	52.414
+ Weigth				133.53	54.51
< None >				105.2	38.772
+ Length		1	5.3794	110.58	40.063
+ Endosperm dying II		1	24.5823	129.79	50.631
+ Weigth		1	30.5177	135.72	53.582

Summary		Estimate	Std. error	t value	Pr( >|t|)
(Intercept)		− 1.84335	0.61352	− 3.005	0.003834**
+ Weigth		0.88634	0.209	4.241	7.55E−05***
+ Length		0.16034	0.09005	1.781	0.079889
+ Endosperm dying II		0.09112	0.02394	3.806	0.000326***
Residuals:	Min	1Q	Median	3Q	Max
	− 3.0946	− 0.7947	− 0.1531	0.4237	2.9798

## Discussion

The large number of unformed seeds in the sample was the first observation that drew our attention. It must be stressed that only the seeds visually wholesome were taken to the analysis. In the case of *Pulsatilla* the collective fruits commonly contain some portion of undeveloped seeds. Selective abortion of lower-quality offspring is found in many species and is intended to increase average progeny fitness^29,30^. According to some authors, in the case of *Pulsatilla* such undeveloped seeds can be simply distinguished from those of full value, e.g. by color, size, and the length of haired styles^27,31^. However, our research contradicts that. It suggests that some of the seeds could be aborted at a relatively late developmental stage. Some authors propose that such a strategy is adopted in the case of plants with seeds destroyed by predators. The bigger number of seeds reduces the impact of pre-dispersal seed predators^32^. Both in Poland and Germany, cases of the destruction of spring pasque-flower specimens by game animals were reported^8^, but these animals remove the whole collective fruits located on long stalks, so a delay in seed abortion could not have made any change. However, there are some reports concerning rodent and insect predators in the case of species from *Pulsatilla* genus^33,34^, therefore, it cannot be excluded that this type of adaptation has also developed in the case of spring pasque-flower. The probability that the large percentage of defective seeds is due to shortage of available resources or pollen limitation^30,35–37^ is low in our case, because of artificial pollination, fertile soil, watering and weed removal in ex situ cultivation.

The number of defective seeds depended on the origin of plants. There were no empty seeds in the case of plants of Alpine origin. This result suggests existence of genetic or epigenetic reasons for the varied seed quality, which is a common pattern^38–40^. In the Alps, *Pulsatilla vernalis* occurs in its core geographical range, in Polish lowlands it grows in isolated localities, where the species survived climatic change. Lowland localities are believed to be relics of the lowland species range from the Pleistocene cold-climate periods^7^. The lowland populations as they are smaller than the Alpine ones, and isolated for a long time, may have been subjected to adverse genetic processes. The analyses performed by Hensen et al.^27^ on the genetic diversity of *Pulsatilla vulgaris* showed that these depended on the population size and were significantly lower in smaller than in larger populations. In the case of the former, a smaller number of ovules developed into fruits, and seeds occurred to be less fertile. These findings are in accordance with the ones presented here: although all the plants cultivated ex situ were grown in the same conditions, those of Alpine origin have more viable seeds. However, it should be remembered that also epigenetic processes regulate seed maturation, dormancy and germination^41^. In our case, e*x situ* cultivation of spring pasque-flower from the seeds coming from the Alps lasted too short to exclude the effects of this type. Different conditions in mountain grasslands could theoretically affect strategies related to the number of aborted seeds, the ability of fully developed seeds to survive winter, etc. Further studies are required to understand differences in life strategies between mountain and lowland populations.

It is worth noting that the stages of embryo development observed within each group of the studied seeds were diverse (Table 1). According to the literature, morphological dormancy means that embryo is underdeveloped in terms of size, but not in terms of its differentiation into cotyledon and hypocotyl-radicle^17,18^. It could mean that during dispersal of the seeds, embryo should be at the torpedo stage. This was confirmed by research on *Pulsatilla vulgaris*, in the case of which torpedo-shaped embryos were found^42^. In the case of *Pulsatilla vernalis* we found a different pattern. Both among those taken from natural site and ex situ collection, there were healthy seeds with embryos at varied developmental stages. It is obvious that the advanced stage of embryo development during dispersal maximizes the seed chances of producing a strong seedling. However, various embryo developmental stages could lead to different germination time. It can be a significant adaptation. In the case of one of Polish lowland populations, which was subject to observation for seven years, strong evidence was found that germination strictly depended on precipitation in August^22^. In lowlands, such weather, with the sufficient amount of moisture, appears irregularly. The relationship between germination time and the degree of embryo development during dispersal needs to be proven in the dedicated studies. Nevertheless, our findings allow to hypothesize that the phenomenon of biological bet-hedging can occur here. Such a strategy was described for plants living in climates with unpredictable rainfalls, especially in arid ones^43–45^, however it occurs in a wide variety of habitats and types of organisms^46^.

The seeds with morphological dormancy (MD) do not require a specific dormancy-breaking treatment i.e., a signal input from external environment to start to germinate^17^. External environmental conditions can influence only the speed of embryo development^20^. In wild lowland populations, the seeds usually mature in June and sprout in August. In our case we have kept the seeds for four months in dry and dark conditions before the laboratory analyses. We stated that 9% of N, 14% of P, and 27% of A seeds had healthful embryos and bigger or smaller number of dying endosperm cells. It can be explained by the usage of endosperm by the growing embryo. Endosperm supports both embryogenesis and germination^47–50^. Of course, external factors can also be responsible for degradation of internal parts of the seed. The selective dieback of endosperm in such a case should be explained by the fact that embryo is hidden deeper inside a seed. However, in our sample there were seeds with a bigger number of cells dying in embryo than in endosperm. The preliminary studies showed no damage of the seed coats of the stored seeds (Appendix 1), and we found important differences between the seeds of different origin despite the same storage conditions. Therefore, it seems very probable that we observed the process typical of MD seeds when embryos grow at the endosperm cost, and such seeds should have a chance to sprout if they were transferred to optimal conditions. The hypothetical chances of germination of these seeds were diversified. The stage of embryo development ranged from globular-heart transition to mild torpedo. The degree of endosperm dieback was also varied, from about 19% of cells at the first stage of dying with the other ones alive, to the N seed with 82% and 18% of endosperm cells at the first and the second stage of dying, respectively. The latter pattern suggests very intensive use of storage material and considering that the embryo in that case was only at the globular-heart transition stage it is difficult to say whether there was enough of it for germination.

A number of seeds (15% of the total number of seeds and 20% of seeds taken to laboratory tests) was classified as degenerating ones assuming that seeds with dying cells in embryo would have had the least chance of germinating. The degeneration of these seeds may have resulted either from their defectiveness at the stage of dispersal, or from the fact that they did not have proper conditions to sprout when their dormancy time ended. Only one seed from that group had approximately half the number of embryo cells dying and endosperm alive at 98%. In the remaining cases, various degrees of endosperm dieback were observed. There were four seeds with torpedo or nearly torpedo stage of embryo, three of them had more than 70% embryo cells alive. In these cases, it can be assumed that if the seeds had been sown without delay, they would have had a good chance of producing healthy seedlings. Four months of storage occurred to be too long. The ‘germination time window’ for these MD seeds closed too quickly.

Morphological dormancy presented by *Pulsatilla vernalis* is thought to be the most primitive dormancy type, typical of primitive angiosperms and some gymnosperms^18^. There is a competitive hypothesis that morphophysiological dormancy (MPD) is the ancestral state of seed plants, but anyway MD remains one of primary dormancy types^51^. Quick loss of seed viability and no environmental clue required to start germination is not favorable for the species in the face of current, rapid climatic changes. At the same time, MD seeds are difficult to preserve in seed banks. *Pulsatilla vernalis* is not adequately adapted to survive in the Anthropocene, at least in the lowlands. However, results show that seeds of *Pulsatilla vernalis* significantly differed. The fact that the width of the aforementioned ‘germination time window’ is varied, can suggest some variations in the dormancy level. According to many studies, such differentiation may be an adjustment strategy of plants growing in unpredictably variable environments^45,52^. Further research on this aspect may be very important for the effective protection of the species under study.

The viability of the studied *Pulsatilla* seeds is related to their weight, and to some extent also to their size, expressed by length (Fig. 5, Table 2). The former parameter is apparently related to their origin, the seeds of pasque-flowers of Alpine origin were heavier. A similar pattern was observed in the case of the other *Pulsatilla* species. Mean seed mass of *P. vulgaris* was attributed to the population size^27^. The relationship between seed mass and the size of *P. vulgaris* population was also found by Pfeifer et al.^53^. The pattern is the same as in our studies even though *P. vulgaris* is not an Alpine-Arctic species with only small remnant populations in Central European Lowlands. Our studies demonstrated that weight is not only connected with the number of alive cells in embryo and endosperm, we found its positive relation to further stages of embryo development (Table 3). The positive relation was also found between the percentage share of alive embryo cells/the embryo development stage, and the share of endosperm dying cells at the second phase of this process. These can be linked to the phenomenon of endosperm digestion by a growing healthy embryo.

## Conclusions for conservation activities

We found greater similarity between the seeds of the same origin than between those from in situ and ex situ cultivation. It can be concluded that ex situ cultivation and controlled pollination do not prevent getting the seeds of quality similar to the quality of the wild ones. Although the use of local-origin seeds is usually a sensible principle in conservation management such as population reinforcement, prompting gene flow between lowland populations could be a strategy preventing the unfavorable genetic phenomena. Similar conclusions were drawn by Gargiulo et al.^54^ concerning *Pulsatilla vulgaris*. However, conservation strategies should not lead to mixing up lowland and mountain genetic pools of spring pasque-flower. The impact of phylogeny and source climate on dormancy and germination of seeds coming from different populations is more and more studied and considered in the restoration activities^55^, and still little is known about the differentiation between mountain and lowland populations of *Pulsatilla*.

Viability analysis of embryo and endosperm cells showed that spring pasque-flower seeds should not be stored. In our case, the four months of storage occurred to be too long. Of course, this does not apply to all seeds, as they vary greatly, but it can be hypothesized that the number of seeds capable of germination decreases with time. More research on proper conditions allowing for embryo maturation and breaking dormancy is needed, especially because the successful reproduction of the species in natural sites in Central European Lowlands is observed only sporadically^2,22^.

It is not always possible to distinguish undeveloped seeds by their external characteristic, however, weight is a good indicator of *P. vernalis* seed value. It correlates with tissue viability and embryo developmental stage.

## Materials and methods

*Pulsatilla vernalis* (L.) Mill. (syn. *Anemone vernalis* L.) is a member of the buttercup (*Ranunculaceae*) family. It is a long living hemicryptophyte with evergreen leaves appearing in rosettes. Each specimen can develop several rosettes lying close to each other and forming compact clusters. Each rosette can give up to three unbranched stems with the leaf whorl underneath flower. Seeds are brown achenes equipped with long-haired style. One flower gives a cluster of approx. one hundred achenes (during our studies we found clusters ranging from 43 to 205 seeds) (Fig. 1).

The research material consisted of *Pulsatilla vernalis* seeds collected from natural site and ex situ cultivation. 22 seeds were collected in June 2019 from a lowland site near the village Dołki in Poland (Świętokrzyskie Voivodeship, Małogoszcz district: E:20°18′30.77″ N:50°48′23.71″), and 44 seeds were taken from plants growing in the Botanical Garden in Łódź (central Poland). Plants grown ex situ were sown in 2015, the seeds were taken from two sites: one was the mentioned locality near Dołki, the other was Col du Galibier saddle in Western Alps (France, 2645 m n.p.m.). Pasque-flowers cultivated in the common garden conditions usually maturate much faster than those in the natural lowland localities, so in 2019 it was possible to collect seeds from six flowering specimens (three of Polish origin and three of Alpine origin). The plants from the botanical garden were cross-pollinated in a controlled manner to obtain seeds representing the genetic lines corresponding to the place of origin. The plants were protected against accidental pollination by means of bags from Duraweld material (produced by PBS International). Summarizing, we analyzed 22 seeds from natural site (in the article we mark them with the letter N), 22 from ex situ cultivation of specimens of Polish origin (letter P), and 22 from ex situ cultivation of specimens of Alpine origin (letter A). The seeds subject to the analysis were visually evaluated as wholesome. Hensen et al.^27^ in the case of quite similar species – *Pulsatlilla vulgaris* – stated that fully developed seeds can be easily distinguished from the undeveloped ones by their different color, and length of feathery styles. The same was said by Bochenková et al.^31^ in the case of *Pulsatilla pratensis*. So, we adopted the same rules to choose the seeds suitable for analysis from the whole set of ovules collected from each specimen. The seeds from the wild population were collected under permission WPN.6400.15.2018 (RDOŚ Łódź). The collection was made by authors in compliance with the relevant legal guidelines and regulations. The repeatability of the research was ensured by the continued maintenance of ex situ plantation in the Botanical Garden in Lodz. The natural locality is protected by law. It is not allowed to collect individuals from natural populations, however, it is possible to obtain permission to collect seeds again.

The seeds were weighed (including the flagella) with semi-microanalytical laboratory balance (A&D model HM-202, accuracy of 0.00001 g). Then they were photographed using a 5.0 MP PRO microscope camera mounted on a binocular. The length of the seeds was measured using the ToupView computer program. Initially, the width was also measured, but it was found that this measure depended on how the seed was photographed (some seeds were flattened).

**Figure 6:**
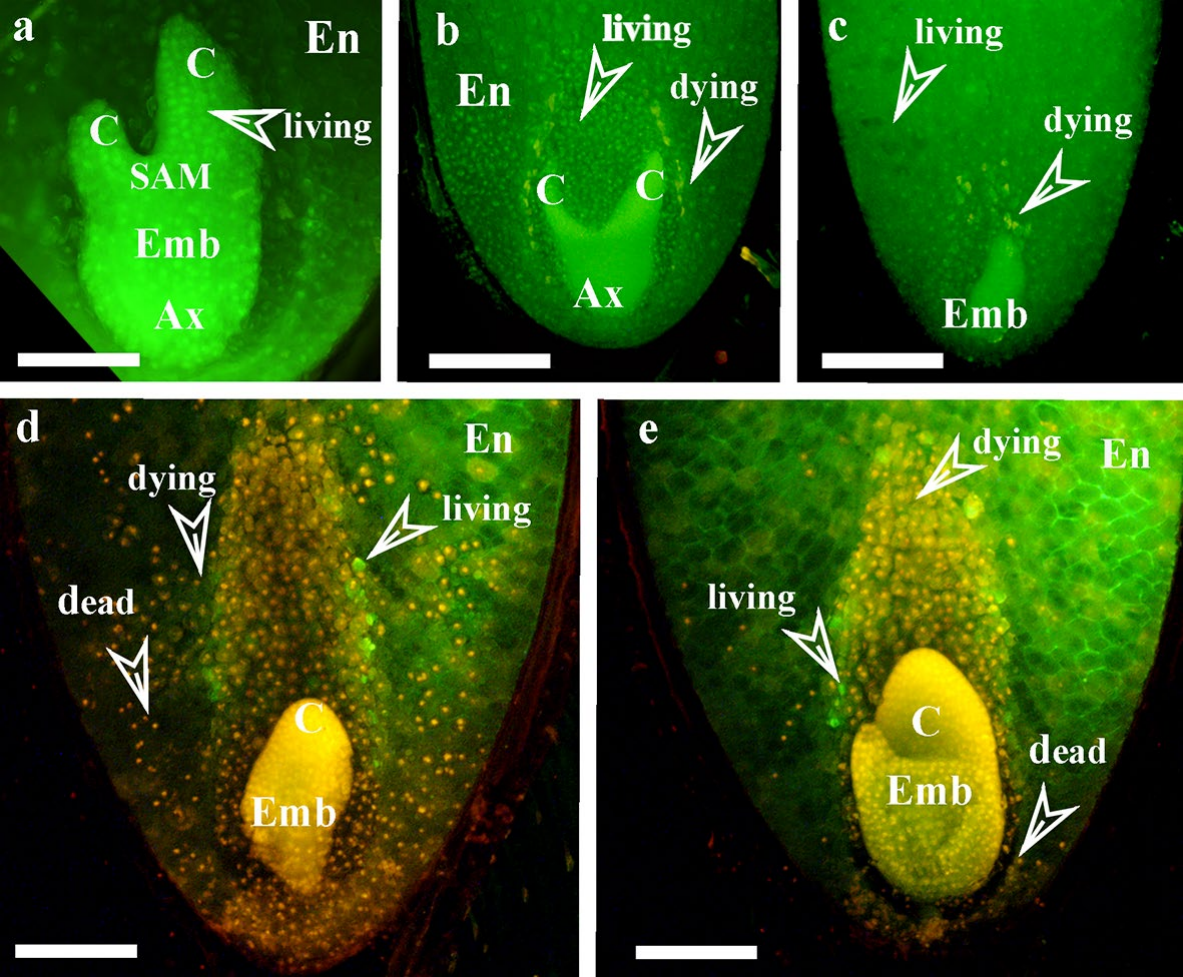
Fragments of cross-sections of *Pulsatilla vernalis* seeds. Living cells (green nuclei) in endosperm (**a**–**e**) and in embryo (**a**–**c**) as well as dying cells (yellow-orange nuclei) and dead cells (red nuclei) in endosperm (**b**–**e**) and in embryo (**d**,**e**) noted under the fluorescence microscopy after staining with ethidium bromide and orange acridine indicated by description and arrows. Scale bars = 100 µm. Ax, axis; C, cotyledon; Emb, embryo; En, endosperm.

Laboratory analysis was conducted four months after seed dispersal. In order to detect cell death the following procedure was adopted, the seeds were: (1) washed twice with 0.01 M phosphate buffer, pH 7.4 (PHB); (2) stained for 5 min with a “staining mixture” containing 100 μg ml^−1^ acridine orange (AO) and 100 μg ml^−1^ ethidium bromide (EB) in PHB, (3) washed twice with PHB, (4) fixed with 2.5% glutardialdehyde (Merck) in PHB for 15 min, and (5) cut with a razor blade along the long axis; (6) then washed twice for 2–3 min with PHB, put on glass slides with a drop of PHB, and analyzed using Optiphot-2 epi-fluorescence microscope (Nikon) with a blue filter (B2A) equipped with a camera (DXM 1200) and Act-1 software (Precoptic, Poland, http://www.precoptic.pl). In this approach, the nuclei of cells with unchanged cell membranes appear green, because they are exclusively labelled by Acridine Orange. With progressive permeabilization of the membrane, the red signal from Ethidium Bromide increases, so that the nuclei convert from green to yellow, orange and finally into red (Fig. 6). The resultant fluorescence intensity (RFI) of the final colors of the chromatin quantified using the Scn Image software (Scion Corporation, open source; http://www.scioncorp.com) allowed classification of the cells into living (green), dying stage I (yellow) and those at dying stage II (orange) and dead (red)^56^.

The CorelDraw Graphics Site X7 EduLic (https://www.coreldraw.com/pl/product/coreldraw) or Inskape (open source; https://inkscape.org/release/inkscape-1.0.1) software were used to prepare figures and images planes.

In order to capture the main dependencies, boxplots and PCA analyses were made using ‘ggpubr’, ‘ggplot2’, ‘ade4’ and ‘factoextra’ packages of R 3.5.2 Statistical Software^57–59^. Since the structure of the data prevented application of ANOVA to determine if the seed weight, length or endosperm state are connected with the percentage of alive embryo cells, the models counted with the usage of multiple stepwise linear regression were used. Step regression procedure was conducted with ‘vegan’ package^60^ after the check of variable colinearity with the variance inflation factor (VIF) with the ‘car’ package^61^. During this analysis we did not take into consideration the percentage of dying and dead cells of embryo, because they are naturally correlated with the percentage of cells that are alive. The same method was used to check which variables are linked to the stage of embryo development. To perform the calculations, numbers from 1 to 4 were assigned to the following successive stages: globular, globular-heart transition, heart, torpedo (to that category we included also the seeds with embryo between the heart and torpedo stages). In the case of seeds without embryo we used the value 0.

## Supplementary Information

Supplementary Information 1.

Supplementary Information 2.

## Data Availability

The data generated or analyzed during this study are included in this article and its Supplementary Information files. The images of cross-sections of *Pulsatilla vernalis* seeds are available from the corresponding author upon reasonable request.

## References

[1] Zielińska KM, Kiedrzynski M, Grzyl A, Rewicz A (2016). Forest roadsides harbour less competitive habitats for a relict mountain plant (Pulsatilla vernalis) in lowlands. Sci. Rep..

[2] Zielińska, K. M., Kiedrzynski, M., Grzyl, A. & Tomczyk, P. P. Anthropogenic sites maintain the last individuals during the rapid decline of the lowland refugium of the alpine-arctic plant *Pulsatilla vernalis* (L.) Mill. *Pak. J. Bot.* **50**, 211–215 (2018).

[3] Grzyl A, Ronikier M (2011). *Pulsatilla vernalis* (*Ranunculaceae*) in the Polish lowlands: Current population resources of a declining species. Pol. Bot. J..

[4] Åström S, Stridh B (2003). The present status of Pulsatilla vernalis in Sweden. Sven. Bot. Tidskr..

[5] Chappuis, E. Pulsatilla vernalis. The IUCN Red List of Threatened Species 2014: e.T55730086A55730098. (2014). 10.2305/IUCN.UK.2014-1.RLTS.T55730086A55730098.en. Downloaded on 02 December 2020>.

[6] Ronikier, M. *et al.* Phylogeography of Pulsatilla vernalis (L.) Mill. (Ranunculaceae): Chloroplast DNA reveals two evolutionary lineages across central Europe and Scandinavia. *J. Biogeogr.* **35**, 1650–1664. 10.1111/j.1365-2699.2008.01907.x (2008).

[7] Kiedrzyński M, Zielińska KM, Kiedrzyńska E, Rewicz A (2017). Refugial debate: On small sites according to their function and capacity. Evol. Ecol..

[8] Betz C, Scheuerer M, Reisch C (2013). Population reinforcement—A glimmer of hope for the conservation of the highly endangered Spring Pasque flower (*Pulsatilla vernalis*). Biol. Conserv..

[9] Nawrocka-Grześkowiak, U. & Frydel, K. Spring pasque-flower (Pulsatilla vernalis (L.) Miller) localities in the Kaliska Forest District. *Zarządzanie Ochroną Przyrody w Lasach* **6**, 77–84 (2012).

[10] Gutterman, Y. In *Seeds: The Ecology of Regeneration in Plant Communities* (ed M. Fenner) 59–84 (CAB International, 2000).

[11] Luzuriaga AL, Escudero A, Perez-Garcia F (2006). Environmental maternal effects on seed morphology and germination in Sinapis arvensis (Cruciferae). Weed Res..

[12] Rao NK, Dulloo ME, Engels JMM (2017). A review of factors that influence the production of quality seed for long-term conservation in genebanks. Genet. Resour. Crop Evol..

[13] Doniak, M., Barciszewska, M. Z., Kaźmierczak, J. & Kaźmierczak, A. The crucial elements of the 'last step' of programmed cell death induced by kinetin in root cortex of V. faba ssp. minor seedlings. *Plant Cell Rep.* **33**, 2063–2076. 10.1007/s00299-014-1681-9 (2014). 10.1007/s00299-014-1681-9 25213134

[14] Doniak M, Byczkowska A, Kaźmierczak A (2016). Kinetin-induced programmed death of cortex cells is mediated by ethylene and calcium ions in roots of Vicia faba ssp minor. Plant Growth Regul..

[15] Doniak M, Kaźmierczak A, Byczkowska A, Glińska S (2017). Reactive oxygen species and sugars may be the messengers in kinetin-induced death of field bean root cortex cells. Biol. Plant..

[16] Tudela-Isanta M (2018). Habitat-related seed germination traits in alpine habitats. Ecol. Evol..

[17] Baskin JM, Baskin CC (2004). A classification system for seed dormancy. Seed Sci. Res..

[18] Finch-Savage WE, Leubner-Metzger G (2006). Seed dormancy and the control of germination. New Phytol..

[19] Latrasse D, Benhamed M, Bergounioux C, Raynaud C, Delarue M (2016). Plant programmed cell death from a chromatin point of view. J. Exp. Bot..

[20] Baskin JM, Baskin CC, Li X (2000). Taxonomy, anatomy and evolution of physical dormancy in seeds. Plant Species Biol..

[21] Grzyl, A. *Biology and Ecology of Isolated Populations of Pulsatilla vernalis (L.) Mill. on the Eastern Limits of its RANGE in Poland*. (PhD thesis. University of Lodz, Department of Geobotany and Plant Ecology, 2012).

[22] Grzyl A, Kiedrzynski M, Zielinska KM, Rewicz A (2014). The relationship between climatic conditions and generative reproduction of a lowland population of Pulsatilla vernalis: The last breath of a relict plant or a fluctuating cycle of regeneration?. Plant Ecol..

[23] Oostermeijer JGB, Vaneijck MW, Dennijs JCM (1994). Offspring fitness in relation to population size and genetic variation in the rare perennial plant species G*entiana pneumonanthe* (*Gentianaceae*). Oecologia.

[24] Ouborg NJ, Vantreuren R (1995). Variation in fitness-related characters among small and large populations of *Salvia pratensis*. J. Ecol..

[25] Fischer M, Matthies D (1998). RAPD variation in relation to population size and plant fitness in the rare Gentianella germanica (Gentianaceae). Am. J. Bot..

[26] Frankham, R., Ballou, J. D. & Briscoe, D. A. *Introduction to Conservation Genetics*. (Cambridge University Press, 2002).

[27] Hensen, I., Oberprieler, C. & Wesche, K. Genetic structure, population size, and seed production of Pulsatilla vulgaris Mill. (Ranunculaceae) in Central Germany. *Flora* **200**, 3–14. 10.1016/j.flora.2004.05.001 (2005).

[28] Jakobsson A, Eriksson O (2000). A comparative study of seed number, seed size, seedling size and recruitment in grassland plants. Oikos.

[29] Melser C, Klinkhamer PGL (2001). Selective seed abortion increases offspring survival in Cynoglossum officinale (Boraginaceae). Am. J. Bot..

[30] Meyer KM, Soldaat LL, Auge H, Thulke HH (2014). Adaptive and selective seed abortion reveals complex conditional decision making in plants. Am. Nat..

[31] Bochenková M, Hejcman M, Karlík P (2012). Effect of plant community on recruitment of Pulsatilla pratensis in dry grassland. Sci. Agric. Bohem..

[32] Ghazoul J, Satake A (2009). Nonviable seed set enhances plant fitness: The sacrificial sibling hypothesis. Ecology.

[33] Laitinen, P. *The Effects of Forest Fires on the Persistence of Pulsatilla vernalis (L.) Mill.* edn, (Ms. thesis, University of Jyväskylä, Faculty of Mathematics and Science, Department of Biological and Environmental Science, 2008) **[in Finnish with an English abstract]**.

[34] Skalická, R., Karlík, P., Hejcman, M. & Bochenková, M. In *17th Symposium of the European Grassland Federation.* 388–390.

[35] Arathi HS, Ganeshaiah KN, Shaanker RU, Hedge SG (1999). Seed abortion in *Pongamia pinnata* (Fabaceae). Am. J. Bot..

[36] Brookes RH, Jesson LK, Burd M (2008). A test of simultaneous resource and pollen limitation in *Stylidium armeria*. New Phytol..

[37] Yang, C. F., Sun, S. G. & Guo, Y. H. Resource limitation and pollen source (self and outcross) affecting seed production in two louseworts, *Pedicularis siphonantha* and *P. longiflora* (Orobanchaceae). *Bot. J. Linn. Soc.* **147**, 83–89. 10.1111/j.1095-8339.2005.00363.x (2005).

[38] Cendán C, Sampedro L, Zas R (2013). The maternal environment determines the timing of germination in *Pinus pinaster*. Environ. Exp. Bot..

[39] Li, R. *et al.* Effects of cultivar and maternal environment on seed quality in Vicia sativa. *Front. Plant Sci.* **8**. 10.3389/fpls.2017.01411 (2017). 10.3389/fpls.2017.01411 PMC5562723 28861096

[40] Valencia-Diaz, S. & Montaña, C. Temporal variability in the maternal environment and its effect on seed size and seed quality in *Flourensia cernua* DC. (Asteraceae). *J. Arid Environ.* **63**, 686–695. 10.1016/j.jaridenv.2005.03.024 (2005).

[41] Chinnusamy V, Gong ZZ, Zhu JK (2008). Abscisic acid-mediated epigenetic processes in plant development and stress responses. J. Integr. Plant Biol..

[42] Butuzova, O. G. Peculiarities of seed formation in *Pulsatilla vulgaris* and *Helleborus niger* (Ranunculaceae) with embryo postdevelopment. *Botanicheskii Zhurnal (St. Petersburg)* **103**, 313—330 (2018) **[in Russian]**.

[43] Duncan, C., Schultz, N., Lewandrowski, W., Good, M. K. & Cook, S. Lower dormancy with rapid germination is an important strategy for seeds in an arid zone with unpredictable rainfall. *PLoS ONE* **14**. 10.1371/journal.pone.0218421 (2019). 10.1371/journal.pone.0218421 PMC6736279 31504045

[44] Gremer JR, Kimball S, Venable DL (2016). Within and among year germination in Sonoran Desert winter annuals: bet hedging and predictive germination in a variable environment. Ecol. Lett..

[45] Venable DL (2007). Bet hedging in a guild of desert annuals. Ecology.

[46] Evans MEK, Dennehy JJ (2005). Germ banking: Bet-hedging and variable release from egg and seed dormancy. Q. R. Biol..

[47] Goldberg RB, de Paiva G, Yadegari R (1994). Plant embryogenesis - zygote to seed. Science.

[48] Lester RN, Kang JH (1998). Embryo and endosperm function and failure in Solanum species and hybrids. Ann. Bot..

[49] Lopes MA, Larkins BA (1993). Endosperm origin, development, and function. Plant Cell.

[50] Yan DW, Duermeyer L, Leoveanu C, Nambara E (2014). The functions of the endosperm during seed germination. Plant Cell Physiol..

[51] Willis CG (2014). The evolution of seed dormancy: Environmental cues, evolutionary hubs, and diversification of the seed plants. New Phytol..

[52] Poisot T, Bever JD, Nemri A, Thrall PH, Hochberg ME (2011). A conceptual framework for the evolution of ecological specialisation. Ecol. Lett..

[53] Pfeifer, E., Holderegger, R., Matthies, D. & Rutishauser, R. Investigation on the population biology of a flagship species of dry meadows: *Pulsatilla vulgaris* Mill. in north-eastern Switzerland. *Bot. Helvet.* **112**, 153–171 (2002).

[54] Gargiulo, R. *et al.* Conservation of the threatened species, *Pulsatilla vulgaris* Mill. (Pasqueflower), is aided by reproductive system and polyploidy. *J. Hered.* **110**, 618–628. 10.1093/jhered/esz035 (2019). 10.1093/jhered/esz035 31102445

[55] Seglias, A. E., Williams, E., Bilge, A. & Kramer, A. T. Phylogeny and source climate impact seed dormancy and germination of restoration-relevant forb species. *PLoS ONE* **13**. 10.1371/journal.pone.0191931 (2018). 10.1371/journal.pone.0191931 PMC5798788 29401470

[56] Byczkowska, A., Kunikowska, A. & Kaźmierczak, A. Determination of ACC-induced cell-programmed death in roots of *Vicia faba* ssp. minor seedlings by acridine orange and ethidium bromide staining. *Protoplasma* **250**, 121–128. 10.1007/s00709-012-0383-9 (2013). 10.1007/s00709-012-0383-9 PMC3557382 22350735

[57] Dray, S. & Dufour, A. B. The ade4 package: Implementing the duality diagram for ecologists. *J. Stat. Softw.* **22**, 1–20. 10.18637/jss.v022.i04 (2007).

[58] Kassambara, A. & Mundt, F. *factoextra: Extract and Visualize the Results of Multivariate Data Analyses*. R package version 1.0.7. https://CRAN.R-project.org/package=factoextra (2020).

[59] Wickham H (2016). ggplot2: Elegant Graphics for Data Analysis.

[60] Oksanen, J. *et al.**vegan: Community Ecology Package*. R package version 2.5-6. https://CRAN.R-project.org/package=vegan (2019).

[61] Fox, F. & Weisberg, S. *An {R} Companion to Applied Regression*, Third Edition. (Sage, 2019). https://socialsciences.mcmaster.ca/jfox/Books/Companion/.

