# Supplementary material for: Short-term cell death in tissues of Pulsatilla vernalis seeds from natural and ex situ conserved populations
Source: Sci Rep. 2021 Aug 19;11:16840. doi: 10.1038/s41598-021-95668-2 (PMC8376884; doi:10.1038/s41598-021-95668-2)
Supplement: Supplementary file 1 — Supplementary Information 1. [file 41598_2021_95668_MOESM1_ESM.pdf]

Short-term cell death in tissues of *Pulsatilla vernalis* seeds from natural and *ex situ* conserved populations

Katarzyna M. Zielińska[1]*, Andrzej Kaźmierczak[2], Ewa Michalska[1]

1. Department of Geobotany and Plant Ecology, Faculty of Biology and Environmental Protection, University of Lodz, Banacha Str. 12/16, 90-237 Lodz, Poland
2. Department of Cytophysiology, Faculty of Biology and Environmental Protection, University of Lodz, Banacha Str. 12/16, 90-237 Lodz, Poland

Appendix 1. Preliminary studies

<u>Germination tests of *Pulsatilla vernalis* seeds</u>

1. Seeds that were collected in 2015 and observed to spring 2016:

Species localities in Poland	Number of sown seeds	Germinated	
		N	%
Dołki	180	61	33.9
Zakrucze	120	88	73.3
Rogowiec	180	52	28.9

2. Seeds that were collected in 2019 and observed to spring 2020:

Species localities and origin	Number of sown seeds	Germinated	
		N	%
ex situ (Alpine origin)	101	16	15.8
ex situ (Dołki origin)	186	5	2.7
in situ Dołki	465	9	1.9

ex situ collection is located in Botanical Garden in Lodz

The samples of seeds from 2019 were taken to laboratory tests described in the article.

3. Seeds that were collected in 2020 and observed to spring 2021 (in that case we had only seeds from *ex situ* collection):

Origin of plants in *ex situ* collection	Number of sown seeds	Germinated	
		N	%
Alpine	57	37	64.9
Dołki	146	23	15.7

Conclusions:

1. Germination of seeds from plants of different origin varies considerably.
2. Germination of seeds collected in different years varies considerably.

Pulsatilla vernalis seeds conductivity testing (Evans blue staining method)

The analysis was performed for seeds taken from *ex situ* collection: 30 seeds of Alpine origin and 90 seeds of lowland origin (Dołki). Conductivity tests were done for samples of 10 seeds.
The table below presents the averages of (1) three measurements in three samples of Alpine seeds, and (2) three measurements in nine samples of Polish seeds.

	after 30 min.	after 60 min.	after 120 min.	after 24 ha
Water conductivity	38.7	38.7	38.7	38.7
Alpine origin seeds	41.9	37.8	47	41.9
Dołki origin seeds	45.5	45.7	48.4	45.5

Conclusion:
No rapid growth at the begining and a significant decrease after 24 ha were observed, we can conclude that seed coats were not damaged.
